# Notch activity opposes ras-induced differentiation during the second mitotic wave of the developing *Drosophila *eye

**DOI:** 10.1186/1471-213X-6-8

**Published:** 2006-02-21

**Authors:** Lihui Yang, Nicholas E Baker

**Affiliations:** 1 Department of Molecular Genetics, Albert Einstein College of Medicine, 1300 Morris Park Avenue, Bronx, NY 10461, USA; 2 Department of Molecular and Cellular Biology, Baylor College of Medicine, One Baylor Plaza, Houston, TX 77030, USA

## Abstract

**Background:**

EGF receptor acts through Ras and the MAPK cascade to trigger differentiation and maintain survival of most of cell types in the *Drosophila *retina. Cell types are specified sequentially by separate episodes of EGFR activity. All the cell types differentiate in G1 phase of the cell cycle. Before differentiating, many cells pass through the cell cycle in the "Second Mitotic Wave" in response to Notch activity, but no cell fates are specified during the Second Mitotic Wave. It is not known how fate specification is limited to G1-arrested cells.

**Results:**

Competence to differentiate in response to activated RasV12 was diminished during the Second Mitotic Wave accounting for the failure to recruit cell fates from cycling cells. Competence was not restored by blocking cell cycle progression, but was restored by reduced Notch activity.

**Conclusion:**

Competence to differentiate does not depend on cell cycle progression per se, but on the same receptor activity that also induces cell cycle entry. Dual effects of Notch on the cell cycle and on differentiation help ensure that only G1 phase cells undergo fate specification.

## Background

EGF receptor acts through Ras and the MAPK cascade to trigger differentiation and maintain survival of most cell types in the *Drosophila *retina [1-6]. Specification of retinal cells occurs as a 'morphogenetic furrow' spreads across the retinal epithelium from posterior to anterior. Because the morphogenetic furrow progresses, each developing eye imaginal disc displays a series of columns of progressively more mature ommatidia posterior to the morphogenetic furrow, beginning with column 0 within the furrow itself[7].

Retinal cells are classified into two groups base on cell cycle behaviour. The first five cells are recruited to each ommatidium during a G1 arrest that begins ahead of the morphogenetic furrow, and these five withdraw from the cell cycle permanently. The remaining cells re-enter the cell cycle within the morphogenetic furrow before being recruited to remaining retinal fates post-mitotically, when they are in G1 phase again[8,9]. The 'Second Mitotic Wave' plays no direct role in specifying or limiting cell fates, but is required to generate adequate numbers of retinal precursor cells[10,11]. It is not known why differentiation is normally restricted to G1 phase cells, given that eye discs contain cells at other cell cycle stages. It is possible that cell differentiation cannot occur in cells actively progressing through the cell cycle, or in cells not in G1 phase. Otherwise a mechanism is required to account for the inverse relationship between cell cycle progression and differentiation.

**Figure 1:**
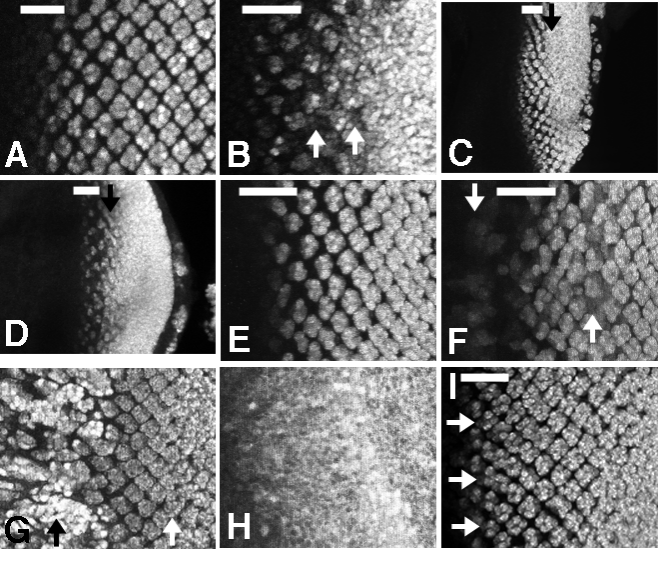
A. ELAV-labelling of differentiating photoreceptor neurons in the eye imaginal disc. Anterior to the left. Bar indicates columns 1–4, where cells progress through the Second Mitotic Wave cell cycle. ELAV protein is first detected in column 2. GMR-GAL4 drives UAS-reporter gene expression in all cells in column 1 and more posteriorly. Progressive addition of neural cells to each ommatidial cluster occurs more posteriorly (rightwards). B. GMR-GAL4>UAS-RasV12. Ectopic neural differentiation is first detected in column 5 (arrows), after the SMW. C. At 29°C, GMR-GAL4>UAS-RasV12 is expected to drive transgene expression at a higher level. Ectopic differentiation is nevertheless restricted until after the SMW, however (arrow). D. RasV12 expression in response to 'strong' GMR-GAL4 likewise affects differentiation only posterior to column 5 (eg arrow). E. 5 h after a 10 min heat shock, hs-RasV12 has not yet affected the pattern of ELAV expression. F. 9 h after a 10 min heat shock, hs-RasV12 has induced ectopic ELAV expression both anterior to the furrow, and posterior to column 7(arrows). G. 14 h after a 10 min heatshock, ectopic ELAV expression (arrows) is observed anterior and posterior to a band of ommatidia that remain little affected. H. More intense Ras activity, due to longer heat shock, leads to general neurogenesis. 14 h after a 1 h heat shock. I. More intense Ras activity, due to transcription from the Sev promoter in SavRasV12, also induces neurogenesis in cells occupying columns 3–5.

The Second Mitotic Wave is centered on columns 1–4 of the developing eye, corresponding to a gap of about 6 h between recruitments of R3/4 cells and of R1/6 cells [11,12](Figure 1A). This gap reflects failure to recruit cycling cells. It is worth reviewing the timing of distinct retinal fate specifications. Within the morphogenetic furrow, the R8 cells that found each ommatidium are specified in column 0[13,14]. R8 cells are recognizable morphologically and beginning to express the neural antigen 22C10 by column 1[15]. R8 cells can be specified in the absence of EGF receptor or Ras activities[2,5,16,17]. Each R8 precursor recruits 4 nearby cells to differentiate as photoreceptors R2, R3, R4 and R5. These and other, later recruitments require EGFR activity[1,2,5]. Several lines of evidence indicate that R2 and R5 have to be specified sometime between columns 0–1, and R3 and R4 at the same time or very soon after. First, cells fated to become R2, R3, R4 and R5 can be identified morphologically in column 1[7]. Their absence from the SMW must be determined before column 2, when all the other cells have entered S phase[11]. R2 and R5 do not express the neural antigen 22C10 until column 3, and R3 and R4 until column 5 or 6[15]. The difference could reflect later specification of R3 and R4, or slower differentiation of R3 and R4. Studies with a temperature-sensitive *egfr *allele show that R2 and R5 differentiation is already EGFR-independent by column 1, although R3 and R4 may remain *egfr*-dependent slightly longer[11,16]. Other cell types are not recruited until after the SMW. In column 4 the first postmitotic cells become available to occupy the niches destined for photoreceptor cells R1 and R6 [7].

How completely can known features of EGFR regulation account for the timing of ommatidial cell recruitment [18]? One factor might be the distribution of EGFR ligands. If R2, R3, R4, and R5 cells need to produce ligands before further cells are recruited, perhaps this does not happen until column 4. Another explanation might be negative feedback. Perhaps levels of the secreted antagonist Argos need to decay before recruitment can resume. If either explanation were correct, cells should be recruited and differentiate prematurely if the EGFR pathway were activated independent of ligands. In contrast to this prediction, expression of activated RasV12 posterior to the morphogenetic furrow leads to ectopic photoreceptor differentiation only after column 5. Prior to column 5, retinal development looks quite normal[11] (Figure 1A,B). Thus, an additional mechanism may be required to explain the pause in retinal differentiation between columns 1–4.

The correlation between the pause in retinal specification and the Second Mitotic Wave suggests that cell cycle progression might be incompatible with cell fate specification. However, evidence from several mutant genotypes indicates that differentiation can also occur in G2 phase, although it is not certain that such differentiation is normal[9,19]. If G1 arrest is not a prerequisite for fate specification, another mechanism must explain why only G1 phase cells are normally selected.

We have investigated why Ras activation posterior to the furrow does not make cells differentiate until after column 5. The experiments indicate that after columns 0–1, cells show reduced competence to differentiate in response to Ras until about column 5. We report that progression through the cell cycle is not the cause, however. Instead we find that Notch signaling activity is partly responsible for preventing differentiation in response to Ras. Based on recent findings that Notch signaling is also essential for S-phase entry in the SMW[20,21], we propose that, in normal development, G1 phase cells differentiate because the Notch activity that promotes cell cycle entry also interferes with differentiation.

## Results

### Reduced competence to differentiate during the Second Mitotic Wave

The GMR-GAL4 driver drives transcription of UAS-trangenes in all eye disc cells posterior to the morphogenetic furrow[1,22]. When GMR-GAL4 drives expression of the activated RasV12, essentially all retinal cells differentiated as photoreceptor neurons, and expressed the neuronal marker ELAV[11](Figure 1A,B). Ubiquitous neural differentiation became apparent around column 6–7, but the pattern and extent of neural differentiation were almost normal earlier, even though GMR-GAL4 driven gene expression is detected from column 1 onwards (our unpublished results). In normal development ELAV protein appeared in R2 and R5 in column 2, indicating a delay of around 2–3 hours between R2/5 specification in column 0–1 and detection of the ELAV protein. The detection of ectopic ELAV expression in column 6–7 suggested that RasV12 was insufficient to recruit ectopic photoreceptor cells earlier than column 5 (Figure 1A,B).

One possible explanation might be that GMR-GAL4 drives UAS-RasV12 expression at a level that is too low to recruit extra photoreceptor cells before column 5. If this were the case, we would expect that higher levels of expression would cause differentiation earlier. To test this we sought to elevate GAL4 activity by raising the temperature. Ras V12 was expressed using the GMR driver at 29°C (Figure 1C). No extra photoreceptor differentiation was observed between columns 1 and 5, although ectopic differentiation still occurred posterior to column 5(Fig. 1C).

RasV12 expression was also increased through use of a distinct GMR-GAL4 insertion (Figure 1D). Previous studies employed a weakly-expressed GMR-GAL4 insertion that does not produce rough eyes when heterozygous[1]. Stronger GMR-GAL4 lines usually have rough eyes in the absence of any UAS transgene, presumably due to 'squelching'(inhibition of genes lacking UAS sites by deprivation of common coactivators by GAL4 [23]). Using a stronger GMR-GAL4 insertion line, for which a single copy leads to a rough eye[24], still did not induce ectopic differentiation before column 6 (Fig. 1D).

To test when cells become competent to differentiate in response to RasV12 in a different way, we exposed all cells to Ras activity simultaneously using the heat shock promoter. Induction using a 10 min heat shock of hs-RasV12 had little effect on differentiation until 9 h later, when ectopic differentiation became visible (Figure 1E–F). Ectopic neurons were seen in the posterior of the eye disc, and anterior to the morphogenetic furrow, but not between columns 1–5 (Figure 1E,F). In addition to monitoring ELAV expression, we also assessed 22C10 antigen, with similar results (not shown). Even 14 h after the heat shock, a region of the eye disc continued to develop normally while ectopic neurogenesis occurred both posteriorly and anterior to the morphogenetic furrow (Figure 1G). These results support the notion that a region around the SMW is more resistant to differentiation in response to Ras activity than are other parts of the eye disc.

Cells in columns 1–5 are not completely insensitive, however, because ectopic neurogenesis was seen there when RasV12 was induced to a higher level by longer heatshock (Figure 1H). In addition, expressing Ras V12 under control of the *sevenless *promoter induces ectopic R7 cells [25]. The Sevenless promoter drives expression at a high level in a dynamic pattern, part of which includes a subset of undifferentiated cells in columns 1–3 ("mystery cells"). We confirmed the observations of Fortini et al[25] that in Sev-RasV12, some ectopic photoreceptors derive from mystery cells in columns 1–3(Fig. 1I). Therefore we conclude that cells require more intense Ras signaling to differentiate in columns 1–5 than at other stages.

### Differentiation blocked independent of cell cycle progression

**Figure 2:**
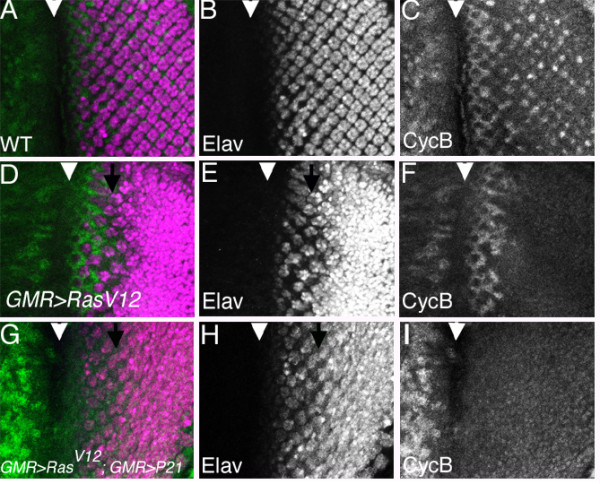
A-C. Normal pattern of neurogenesis and cell cycle progression in GMR-GAL4. Cells enter S-phase of the SMW after column 0, and most cells (~90%) perform mitosis and degrade their Cyclin B between columns 3–5. Arrowhead indicates column 0 in the morphogenetic furrow. D-F. In GMR-GAL4>RasV12, ectopic neurogenesis occurs after the Second Mitotic Wave is completed (arrow). 100% of SMW cells perform mitosis and degrade Cyclin B in this genotype[11]. G-I. GMR-p21 prevents entry into the SMW, so that no cells contain Cyclin B posterior to the morphogenetic furrow [10]. Ectopic neurogenesis is still delayed until after column 5, however (arrow).

One possibility was that differentiation in response to Ras depends on cell cycle status. In normal development, retinal cells differentiate in G1 phase. During columns 1–4 undifferentiated cells are cycling through S, G2 and M phases of the cell cycle (Figure 2A–C). Cells that differentiatiate from column 5 onwards in response to ectopic Ras have completed the SMW cell cycle (Figure 2D–F). If cells were resistant to Ras-induced differentiation due to their cell cycle status in columns 1–4, their competence to differentiate should be restored if cell cycle entry were prevented. RasV12 was expressed in the GMR-p21 background, where cyclin E function is prevented posterior to the morphogenetic furrow and no Second Mitotic Wave occurs[10]. If cell cycle progression interfered with differentiation, we would expect more differention in the absence of cell cycle progression. The effect of GMR-GAL4>RasV12 was little changed by p21 expression that blocked cell cycle entry, however (Figure 2G–I). Ectopic differentiation occurred only posterior to column 6, even though all cells posterior to the morphogenetic furrow remained in G1 (Figure 2G–I). Cells in columns 1–4 must be resistant to Ras for a reason other than cell cycle progression.

**Figure 3:**
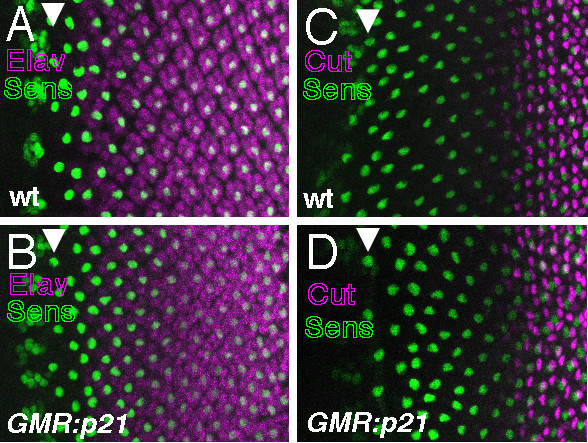
A. In wild type, photoreceptor neurons (ELAV, magenta) differentiate clustered around central R8 cells (SENSELESS, green). Arrowhead indicates column 0 within the morphogenetic furrow, where single R8 cells are first individually resolved. Anterior to the left. B. In GMR-p21, photoreceptor differentiation is first observed in column 2, as in wild type (compare panel A). C. In wild type, cone cells (CUT, magenta) are first detected around ommatidia in column 10. D. Cone cells are detected no earlier in GMR-p21, despite not having to divide before differentiating.

We also checked whether cell cycle progression affected the timing of differentiation in response to endogenous Ras activity (Figure 3). Regardless of the presence of the SMW in wild type or its absence in GMR-p21, the first ELAV expression by photoreceptor cells was seen in column 2 (Figure 3A,B), and the first CUT expression by cone cells was seen in column 10 (Figure 3C,D). Cone cells were monitored as a cell type that normally differentiates only after the SMW[7]. Thus, cell cycle progression did not delay differentiation in response to activity of either endogenous or ectopic Ras.

### Endogenous Notch activity opposes differentiation

One pathway that could interfere with differentation was Notch signaling. Reduced Notch function leads to ectopic neurogenesis [26]. Some of the ectopic neurogenesis might depend on Notch indirectly, because supernumerary R8 cells develop when Notch is reduced and might recruit excess other photoreceptor cell types[27]. Notch can interfere directly with recruitment of cell types other than R8 when constitutively activated, however, and there is evidence that endogenous Notch may do so also [28,29].

**Figure 4:**
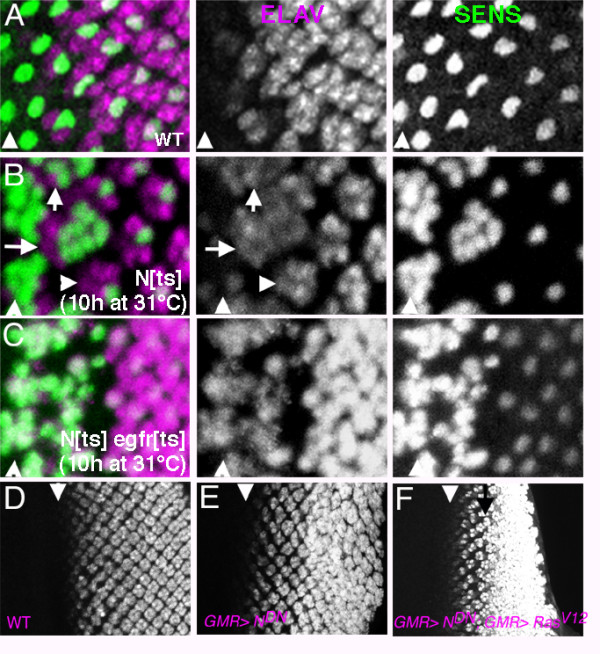
A In wild type, photoreceptor neurons (ELAV, magenta) differentiate clustered around central R8 cells (SENSELESS, green). Arrowhead indicates column 0 within the morphogenetic furrow, anterior to the left. B. Reduced N function causes recruitment of additional neurons (ELAV, magenta). Not all label with the R8 marker Senseless(green). Many are associated with large clusters of R8 cells (arrows). Additional neurons are also recruited by ommatidia that contain single R8 cells, however (arrowhead). Such recruitment reflects N function after R8 cell patterns has been finalized in column 0. C. Ectopic non-R8 neurons are EGFR dependent. When *egfr *function is reduced in addition to *N*, the only extra neurons are R8's. D-F. Ectopic expression on N-DN does little to interfere with normal neurogenesis, or with ectopic neurogenesis in response to activated RasV12.

If N repressed photoreceptor differentiation in columns 1–4, we would expect ectopic neurogenesis in the absence of N function. The temperature-sensitive allele *N*^*ts1 *^was used to test whether ectopic neurogenesis occurred independently of R8 specification. After 10 hr at the restrictive temperature (31°C), Senseless expression revealed differentiation of ectopic R8 photoreceptors from cells that had been anterior to column 0 at the time of the shift, as expected from role of Notch in repressing R8 cells (Fig. 4A,B) [14]. ELAV expression revealed ectopic recruitment of non-R8 photoreceptors around these R8 clusters (Fig. 4B). In addition, occasional extra ELAV-expressing photoreceptors were observed to the posterior, associated with clusters that contained only single R8-like cells labelled for Senseless (Fig. 4B). No such ectopic photoreceptor cells are ever observed in wild type discs. They indicate a normal role of Notch antagonizing differentiation of non-R8 photoreceptor cells even after R8 specification has already occurred[29](the origin of the many extra photoreceptors in more anterior columns cannot prove a direct role of Notch, because there are multiple R8 cells in these ommatidia). This role was limited to a single ommatidial column, however, corresponding to ommatidia around column 0–1 at the time N function was lost(Fig. 4B). Consistent with this, GMR-driven expression of dominant-negative Notch posterior to column 1 did not induce ectopic differentiation of R2-R5 cells, even in the presence of ectopic RasV12, although additional neurons differentiated later, after the SMW (Fig. 4D–F).

If Notch prevented photoreceptor differentiation by antagonizing Ras activity, Ras activity should be required for ectopic photoreceptor differentiation when Notch activity was reduced. An alternative was that the normal role of Ras might actually be to block inhibition due to Notch. In this case Ras would be dispensable for photoreceptor differentiation in the absence of N. *N*^*ts *^*egfr*^*ts *^eye discs were examined to distinguish the models. All photoreceptor cells differentiating in *N*^*ts *^*egfr*^*ts *^eye discs at restrictive temperatures also expressed the R8 marker Senseless, indicating that other photoreceptor cell types required EGFR signaling for differentiation, even in the absence of Notch (Fig. 4C). Taken together, these findings indicate that Notch antagonized Ras activity in some cells in columns 0–1. Notch did not seem required to prevent differentiation by cells in columns 2–4, however.

**Figure 5:**
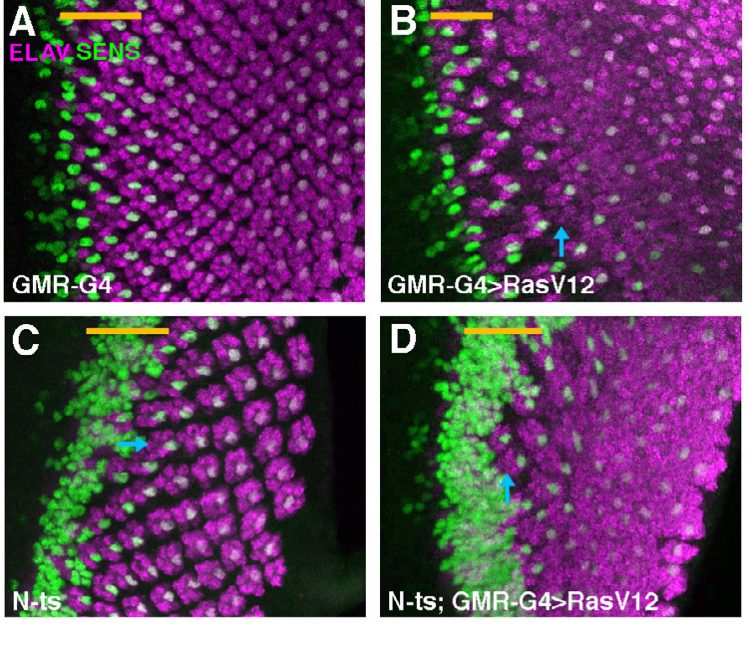
A. In wild type, photoreceptor neurons (ELAV, magenta) differentiate clustered around central R8 cells (SENSELESS, green). Yellow bar indicates columns 1–4, corresponding to the Second Mitotic Wave. B. GMR-RasV12 promotes ectopic neurogenesis posterior to column 5 (eg arrow). Between columns 2–4 most ommatidia (72%) had the normal number of ELAV-positive cells, and only 0.50 extra neurons per ommatidium were seen on average. C. Reduced N signaling results in ectopic R8 specification within the morphogenetic furrow. An average of 0.36 extra neurons were seen in column 4 where R8 cells were unaffected(eg arrow; see also Figure 4B). 68% of the ommatidia in column 4 had the normal number of ELAV-positive cells. There are many more ectopic neurons anteriorly, but as columns 2–3 contained many R8 cells it is unclear how many other neurons differentiated as a direct consequence of loss of N function and how many were recruited by R8's. D. GMR-RasV12 promotes more and earlier ectopic neurogenesis when *N *function is reduced (compare panel B). Arrow indicates extra ELAV-positive cells around ommatidia with single R8 cells. There were 4.1 extra ELAV positive neurons per ommatidium, on average, and in some preparations all the cells appeared to be ELAV positive.

Although it was possible that another pathway prevented differentiation of cells in columns 2–4, another possibility was that Notch was dispensable because there was little EGFR activity in columns 2–4. Since Notch appeared to antagonize the EGFR pathway (Figure 4C), losing Notch function might have little effect where the EGFR pathway was also inactive. In this case one would expect cells to differentiate in response to EGFR pathway activity, if N function was also reduced. RasV12 was expressed in *N*^*ts *^eye discs to test this notion. Either RasV12 expression or reduced N function by themselves led to less than 1 extra neuron per ommatidium in columns 2–4 (Figure 5A–C). By contrast, GMR-GAL4>RasV12 promoted much greater neuronal differentiation in columns 2–4 when N function was reduced (Figure 5D). There were 8 times as many extra neurons on average, and in some preparations almost all available cells appeared to be differentiating. Thus N signalling contributed to cells' resistance to differentiation in response to activated Ras in columns 1–5. In normal development both N signaling and downregulation of Ras activity limit differentiation during the Second Mitotic Wave, which can occur if both are disrupted. Such differentiation must occur in G1 phase, because without N signaling cells do not enter the SMW cell cycle[20,21]. We were unable to test whether N was required to restrict the response of cells to hs-RasV12, apparently because of persistent hs-RasV12 activity at the restrictive temperature for *N*^*ts*1 ^(data not shown).

## Discussion

During normal eye development there is a pause in the progressive recruitment of retinal cells. Between columns 1–4, unspecified cells pass through a cell cycle called the Second Mitotic Wave. Because no fates are specified during this period, differentiating retinal cells are normally all in G1 phase. Even activated RasV12 was not sufficient to drive photoreceptor differentiation until after these cells have completed the cell cycle. One hypothesis was that cell cycle progression precludes differentiation.

We found that cell cycle progression was not required to prevent differentiation, because preventing the SMW did not restore competence to differentiate in response to RasV12 (Figure 2). Preventing the SMW also does not affect the amount or timing of differentiation in response to endogenous Ras[10](Figure 3, 4). Instead reduced competence must in part be due to activity of the receptor protein Notch, because reducing Notch activity increased competence to differentiate in response to Ras (Figures 4,5). In normal development Notch is most critical in column 1, perhaps reflecting declining endogenous Ras activity in columns 2–4. Thus differentiation is limited both by the activity of N and tight regulation of Ras, and perhaps other mechanisms in addition[30].

The role of Notch in reducing competence to differentiate in response to Ras suggests a mechanism that correlates cell fate specification with cell cycle withdrawal. Recently it was reported that Notch activity is required for cells to enter S phase of the SMW[20,21]. We propose that Notch opposes differentiation independently of its cell cycle effects, so that the SMW coincides with a pause in differentiation because both are responses to activity of the same receptor. Ras also has cell cycle effects that have been previously noted. Ras activity blocks S phase entry in column 0, and promotes G2/M progression in G2 cells in the SMW[6]. Ras therefore both promotes G1 arrest of differentiating cells, and return to G1 (via mitosis) of cells that receive EGFR signals in G2.

Previously, EGFR activity has been thought to be determined by the sequential expression of ligands and by negative feedback mechanisms[18]. As each photoreceptor cell type begins differentiating, it becomes a source of EGFR ligands and so expands EGFR activity to new cells. Each round of EGFR activation triggers a peak of negative feedback, which rapidly confines the spread of differentiation at each step. Because expression of the Notch ligand Delta is regulated by EGFR[20,31], Notch activity may be considered a further feedback control on EGFR, acting downstream of Ras. In this view N activity in columns 1–4 is partly a response to earlier EGFR activity in R2-R5 cells, albeit indirectly. Consistent with this, expression of E(spl) bHLH proteins is lost posterior to the morphogenetic furrow in cells mutant for EGFR[5].

It is important to point out that the model we propose is based on a particular stage of retinal development. Although an inverse correlation between differentiation and proliferation is observed in many developmental events[32], we do not yet know whether any mechanism links Notch signaling to proliferation in general, although this is one possibility. Even within the retina, Notch later has roles that promote differentiation of R7 and R4 cell types that appear unrelated to cell cycle progression [33-36].

It is probable that cell cycle and cell specification pathways diverge at the level of target genes of the Notch and Ras pathways. *Dacapo *has been suggested as a component of the G1 arrest program in response to Ras, but even mutating *dap *and *Rbf *simultaneously, which permits cells to continue proliferating, does not block differentiation[21]. One target of Ras signaling in R1, R6, and R7 differentiation is Phyllopod. Phyll is not required for R2-5 specification, although it appears to be expressed there and so might act redundantly [37-39]. Notch target genes mediating S-phase entry or antagonizing differentiation of cells other than R8 remain to be identified. One question raised by our present work is how Notch signaling can be epistatic over Ras activity during the SMW, when earlier EGFR is epistatic over N in preventing cell cycle entry by R2,3,4 and R5 cells and promoting their differentiation [21].

## Conclusion

Normal retinal cells always differentiate in G1 phase of the cell, although the retina also contains actively cycling cells. We report that the cycling cells are resistant to Ras-induced fate specification, but not because they are cycling. Instead, the correlation occurs because the SMW occurs during a pause in cell fate specification, maintained in part by the same Notch activity that drives cell cycle entry, so that cells that enter the cell cycle because of N also resist specification.

Metazoan genomes encode many thousands of genes, whose interactions might potentially be very complex. By contrast there appear to be only a limited number of extracellular signaling pathways that are important for development[40]. This constraint may be enough to make it common for correlated developmental processes to be linked through dependence on a shared extracellular signal.

## Methods

Fixation and immunochemical procedures were as described previously[5,6]. Fly work was conducted at 25°C except where otherwise indicated in the text. Anti-elav, anti-Cut, anti-cyclin B and mAb22C10 antibodies have been described previously [39,41-43]. UAS-RasV12, Sev-RasV12, hs-RasV12, UAS-N^DN^, GMR-p21 and GMR-GAL4 transgenes are all transcriptional fusions of the indicated promoters to the RasV12, N, p21 or GAL4 coding regions. The UAS-N^DN ^line expresses a protein deleted for the intracellular domain [44]. Transgene insertions were heterozygous in all our experiments (the GMR-p21 samples were heterozygous for two independent GMR-p21 insertions) *N*^*ts*1 ^and *hs-*RasV12 flies were obtained from R. Cagan[26,45]. UAS-RasV12 flies were obtained from F. Karim[46]. *Egfr*^*ts*2 ^was obtained from K. Moses[16]. *Sev-*RasV12 was obtained from M. Fortini[25]. GMR-GAL4 flies were obtained from S.L.Zipursky and M. Freeman.

## Authors' contributions

LY contributed the data shown in Figures 1, 2, and 4, NEB the data shown in Figures 3 and 5. Both authors read and approved the final manuscript.
